# After-Treatment of Puerperal Women

**Published:** 1883-09

**Authors:** John M. Keating

**Affiliations:** Visiting Obstetrician to the Hospital


### CLINICAL LECTURE ON THE AFTER-TREATMENT OF PUERPERAL WOMEN. Delivered at the Philadelphia Hospital by JOHN M. KEATING, M.D., Visiting Obstetrician to the Hospital.

Reported by W. A. EDWARDS, M.D.

GENTLEMEN.—Last week a woman entered the hospital, a few days after her confinement, in an apparently healthy condition.

In forty-eight hours, however, local cellulitis had developed, with excessive pain on pressure, tympany, and a rapid rise of temperature. In other words, puerperal fever had set in, supposed to be due to the absorption of infectious material from the uterine surface, the patient having suffered an acute laceration of the cervix in the confinement. This rapid rise of temperature, the attack setting in so soon after labor, and, withal, the onset being so sudden, with obstinate vomiting, pain, tympanitic distention of intestines, interference with respiration, and the rapid sequence of symptoms which all foreshadowed a fatal result, made her condition indeed difficult to treat. It is here, in a case like the present, that I would most strongly advocate antisepsis and even intra-uterine injections.

Dr. T. Gaillard Thomas presents an article in the *New York Medical Journal* for March 31, 1883, recommending these intra-uterine injections in puerperal septicæmia. His cases are certainly extraordinary, and if they did not come from such authority I should be inclined to doubt the accuracy of the observation. One case was delivered in a perfectly normal manner, without difficulty or assistance. The temperature at the end of the fourth day had risen to 106.5°, and the patient presented all the appearance of puerperal poisoning. There was extensive bilateral laceration of the cervix. The uterus was then washed out with carbolized water every four hours, and the temperature at once fell to 101°; ten grains of quinine were given every eight hours, enough being administered to negative the idea of the high temperature being due to malaria. As soon as the temperature regained 105°, the washings were again ordered and the temperature was reduced in like manner.

Dr. Thomas also remarks that the time has arrived when we should treat puerperal septicæmia upon just as simple a plan as septicæmia of any other kind, by washing the surface where the disease originates with some antiseptic fluid. I desire to lay particular stress upon the temperature record in cases of this nature; it is the guide-post to prognosis. The fatality of an epidemic or of an individual case is in proportion to the excessive temperature. It is a fact that prolonged high temperature will undoubtedly kill; this is seen in no other disease more decidedly than in the one under consideration.

During my term in this house, some ten years ago, our obstetrical wards were visited by a severe epidemic of septicæmia, introduced by a scarlet fever patient. I preserved the record of forty cases of high temperature, which all presented much the same symptoms, developing tenderness in the abdomen in a short time after delivery, and in twelve hours the tympanitic distention of the bowels appeared. This abdominal distention is very characteristic, the women looking not unlike the cases of abdominal dropical effusion which you so frequently see tapped in this clinic-room.

The evening temperature generally was 104°–105°, commonly followed by a chill, with marked signs of impaired circulation, difficult breathing, dyspnœa, local pain, more particularly noticed on pressure; and death usually occurred gradually, in about forty-eight hours, from interference with the circulation; the consciousness was preserved until almost the last moment. In most of these cases you will be able to trace this appalling condition of affairs to a local sore, lacerated cervix, and the introduction of septic material. Of course, in this house the conditions are more favorable for its development; the buildings are old, saturated with germs, and the obstetrical wards but little removed from large surgical rooms which are simply reeking with bacteria, micro-organisms, etc., which are but too ready to be carried across the nominal boundary-line. You will thus readily see that the hospital accoucheur is liable to meet more of these cases than in private practice. In fact, it is rare, in an ordinary normal labor in private practice, with no laceration or tearing, and when the forceps have not been used, to meet with excessively high temperature after delivery. From the foregoing remarks you will readily appreciate that the practical point of this lecture hinges upon the *treatment*.

First and foremost, I believe that many of the cases that we hear of septic trouble are due to meddlesome midwifery; for I venture to say that very many of the forceps cases are meddlesome midwifery. I advance this statement from actual observation. I am also convinced that forceps are too much used, and that they are frequently had recourse to not so much for the patient's weal as for the doctor's convenience.

In primiparæ, owing to various causes, too rapid labor may occur, the head passing the os before it is fully or widely dilated, and in consequence a laceration is produced, which directly favors the entrance of septic matter and its attendant train of evils.

Let me impress upon you the fact that you hold the lives of your puerperal patients literally in your hands, and that the practitioner cannot be too careful regarding cleanliness, especially as a busy man may be tempted to go at once from a scarlatinous patient, for example, to a labor case, when he may in all probability encounter an adherent placenta, leading him to introduce his hand, teeming with disease-germs, into the womb, and laying the patient open to grave danger of blood-poisoning.

Listerism shows marked results in reducing the prevalence or liability of septicæmia, though in my opinion the nail-brush, soap, and water are quite as valuable as carbolic acid, if used frequently enough. I must confess that puerperal fever is too often simply another and high-sounding name for carelessness. I do not advocate the delivery of women under the spray, nor am I satisfied as to the efficacy of carbolic acid unless carried down and laid directly on the septic centre.

*Causes.*—Septic material and a denuded surface for its entrance, as an abrasion of the mucous membrane, represent the slow-match and the train. In primiparæ this mucous tissue will stretch but little; longitudinal vaginal tears or rents are therefore frequently seen, and in many cases laceration of the cervix occurs. We then have the most favorable condition offered for the production of septicæmia and hyperpyrexia: as the cervix is high up, it requires more attention in order to keep clean, and the anatomy of the part is particularly favorable to the absorption of any matter in contact with it.

For the prevention of this undesirable condition I will mention three means:

First, prevent the access of septic matters to denuded tissues by cleanliness.

Second, make proper application of antiseptics to local abrasions.

Third, administer drugs which act directly on the uterine tissue, preventing the absorption of septic material by causing contraction.

After labor you should find the womb firm, hard, and globular; after a time it will relax, become larger, the cavity opens, and the sinuses again become patulous. It is now that the septic material gains entrance and starts on its death-dealing path. Here we find the indication for drugs.

Fordyce Barker some ten years ago insisted upon keeping the womb firmly contracted, and showed very conclusively the value of ergot, nux vomica, quinia, and iron,—in fact, of all drugs which contract tissue and act on unstriped muscle-fibre.

You will find great diversity of opinion in regard to the action of these drugs; for example, some claiming for quinine a powerful oxytocic aetion, others stoutly denying it, and advancing as an argument the fact that Southern women while pregnant take large quantities of the drug, made necessary by their malarial climate, without any bad effects.

A paper published in France in 1872 or 1873 probably gives us the true explanation of the action of these two substances. By experiments on animals it was found that ergot did act upon the uterus, provided that it was determined to that organ by some irritation, and if this irritation did not exist it would not act. So with quinine. The conditions are the same in the human female as in the animal with the artificially-irritated womb. We thus find that ergot and quinine will be determined to the organ, in the case of the former, at all events, producing decided contraction. High temperature is a danger in itself, as it produces acute fatty degeneration of the cardiac and uterine muscular fiber and of that of the intestinal canal; in fact, all the essential muscles succumb to this pathological action; as a result we have meteorism, attended by its disastrous consequences that I have already referred to. The womb in this condition is unable to contract. The knowledge of this fact is, I take it, the touchstone in guiding the case to a successful termination, and this point escapes many in their after-treatment of these cases. Some authorities say that this degeneration is due to fever-poison coursing through the circulation, as seen in the typhoid fever heart; but, according to my interpretation, hyperpyrexia is the root of the trouble.

Death will rapidly occur, occasionally from excessive meteorism, but generally from extreme prostration; and, as this latter condition is due to a burning up of the tissues, the important indication is to depress rapidly the temperature, prevent absorption from abraded surfaces, secure proper contraction of the womb, and relieve local symptoms. First and foremost, we should aim to secure an abundant supply of pure fresh air, make the patient as comfortable as possible under the circumstances, and administer full doses of quinine as an antipyretic and for its beneficial tonic action on the nervous system, as we know the uterus is so dependent on nerve-influences, and, furthermore, as the contractions are entirely involuntary. You may also use cold applications, spraying, etc., or the cold coil, which is so popular with English accoucheurs.

I usually, especially in this house, make it my routine practice to administer quinine and ergot immediately after labor, believing, as I do, that they exert a specific action on the womb.

I also deem it expedient to exhibit small doses of light cathartics, more especially when we are treating high temperature, a dry coated tongue, etc. Calomel is usually selected, in doses of of $\frac{1}{16}$ $\frac{1}{12}$ $\frac{1}{8}$ gr., rubbed up with sugar of milk, and taken every half-hour until a decided action on the bowels is secured, when the thermometer will register a marked fall in the temperature and your patient will feel much more comfortable. I always administer this calomel treatment, even after we have sewed up up the perineum, for example. I do not at all advocate putting the bowels "in a splint," as our patients are decidedly more comfortable by having a daily evacuation, and, furthermore, there is less chance of tearing out the stitches. It is my practice to require the bowels of a parturient female to be opened before labor; but this, of course, is difficult to regulate in private practice.

As I have already told you, an important indication is to give drugs which will promote uterine contraction. The following I find is the most acceptable way to administer them :

| | | |
|---|---|---|
| ℞ | Ext. ergotæ fl.................................... | f℥iii |
| | Tinct. nucis vom................................. | f℥ss |
| | Tinct. ferri chloridi........................... | f℥ii |
| | Acid. muriat. dil.............................. | f℥i |
| | Syr. limonis.................................... | |
| | Aquæ, āā q. s. ad.............................. | f℥vi |
| M. S. | | |

This is usually an unpleasant mixture ; but if you direct that the acid and iron be first added together, and then the nux vomica, you will prevent formation of the tannate of iron, and consequently the inky taste.

For the same motive,—namely, to promote uterine contraction, I am a firm advocate of the binder, especially in a loose, flaccid abdomen following great stretching, and also of the use of turpentine externally. The irritation of constant pressure will secure contraction and prevent the gradual enlargement of the womb.

*The Use of Vaginal Injections* —This is the most important part of my lecture to-day. Dr. Thomas found, as I have already mentioned, that injections of carbolized water into the uterine cavity would reduce the general bodily temperature three to four degrees. You must use a Chamberlain tube which is of large calibre and will not allow the fluid to be introduced directly into a uterine sinus, the consequence of which would, as you know, be disastrous to your patient. If, however, the laceration is farther down in the sexual canal, you must be careful that your nurse does not simply wash the septic matter up into the womb and imprison it there : hence direct her to introduce the syringe high up, so that the water in finding its way to the external parts shall carry all septic material with it.

Personally, I do not ordinarily use syringes in private practice, and I do not meet with any higher temperature than those who use them in the old fashioned way. I depend on securing tonic contraction of the womb by firm external pressure and reflex irritation, and simply order the nurse to wash the patient thoroughly with an antiseptic solution. For this purpose I use an old-fashioned remedy,—*i.e.*, alcohol in which is dissolved as much castile soap as is possible: this is soft, agreeable, and thorough.

In France they use solutions of corrosive sublimate as a wash, also intra-uterine suppositories of iodoform. This has reduced temperature as effectually as Thomas's carbolized water. There are also various antiseptic washes, but I will not occupy your time with them: the principle upon which they all are used is, as you know, their toxic action upon the lower forms of life.

If you reflect for an instant, you will see that the walls of the parturient canal after labor are once more approximated, and air does not enter. There is little chance for the accumulation of discharges, if you direct your patient to move about. I do not at all believe in allowing our puerperal patients to lie flat on their backs; you must order that they move about in the bed, turn from side to side, and thus relieve the canal of clots and discharges. Position, thus, to some extent, takes the place of injections. Hence I would impress upon you not to use the injection mechanically, and not to order it because it is fashionable; see to it that the nurse does not do more harm than good. Watch the heart and pulse, that their strength may be kept up and the temperature reduced. You must be prepared to associate digitalis with the medicinal treatment as soon as any tendency to heart-failure is manifest.

In some text-books, but more particularly in journals, aconite and veratrum viride are recommended in cases of puerperal fever. I warn you about these cardiac depressants. Do not let these journal articles cloud your reason; it is only in the early stages of the disease, and more especially in puerperal peritonitis, that you are at all justified in prescribing these drugs, and thus bleeding the patient into her own body, just as leeches would do externally; it is only in a typical sthenic case, in fact, in a case in which the older practitioners would bleed from the arm, that you are at all justified in exhibiting these depressants, and then my preference would be for aconite. Some, however, recommend veratum viride. As a rule, these cases are asthenic, when the general plan of treatment must be stimulant, both general and cardiac. The study of the *vomiting* is also of great importance.

It is most obstinate and appalling in private practice; you will frequently meet with cases who cannot tolerate even a teaspoonful of pure cold water. This vomiting is of pneumogastric origin, due to the irritation of the remote fibres of the nerve found in the sexual organs.

Drugs must now be administered by the hypodermic syringe, or by rectal suppositories. Quinine may be given by the former method; there is little danger of producing abscesses, provided the solution be perfect and the needle deeply introduced into the muscular fibre.

Opium is indicated, and may be introduced by either the latter or the former method. The indication is, of course, the relief of pain or vomiting, quelling the nervous excitement, and as a tonic to the nervous system. If the pain is local and low down, a suppository is more efficacious; otherwise a hypodermic of morphia and atropia, small doses of calomel, dilute hydrocyanic acid, or complete rest of the organ may relieve the vomiting.

To secure the requisite amount of nourishment for your patient is, of course, difficult under these circumstances. Milk and lime-water frequently repeated and in small quantities is efficacious, also brandy and frozen beef-tea, or beef-juice, thus relieving thirst and supplying food at one and the same time. Koumiss will sometimes be retained when milk will not. Carbonic acid water added to the milk makes it light and palatable. Iced champagne will refresh and relieve the patient. You must use free feeding and stimulation.

I know of no more appalling death that you will witness than one of puerperal septicæmia, the patient gradually dying of exhaustion and from fatty degeneration of the heart-muscle, with consciousness fully retained until the last scene in the drama of life is over.—*Medical Times.*

